# Investigating the Impact of a Curse: Diseases, Population Isolation, Evolution and the Mother’s Curse

**DOI:** 10.3390/genes13112151

**Published:** 2022-11-18

**Authors:** Maria-Anna Kyrgiafini, Themistoklis Giannoulis, Katerina A. Moutou, Zissis Mamuris

**Affiliations:** 1 Laboratory of Genetics, Comparative and Evolutionary Biology, Department of Biochemistry and Biotechnology, University of Thessaly, Viopolis, Mezourlo, 41500 Larissa, Greece; 2 Laboratory of Biology, Genetics and Bioinformatics, Department of Animal Sciences, University of Thessaly, Gaiopolis, 41336 Larissa, Greece

**Keywords:** mother’s curse, mitochondrion, mtDNA, mito-nuclear incompatibility, evolution, population genetics

## Abstract

The mitochondrion was characterized for years as the energy factory of the cell, but now its role in many more cellular processes is recognized. The mitochondrion and mitochondrial DNA (mtDNA) also possess a set of distinct properties, including maternal inheritance, that creates the Mother’s Curse phenomenon. As mtDNA is inherited from females to all offspring, mutations that are harmful to males tend to accumulate more easily. The Mother’s Curse is associated with various diseases, and has a significant effect on males, in many cases even affecting their reproductive ability. Sometimes, it even leads to reproductive isolation, as in crosses between different populations, the mitochondrial genome cannot cooperate effectively with the nuclear one resulting in a mito-nuclear incompatibility and reduce the fitness of the hybrids. This phenomenon is observed both in the laboratory and in natural populations, and have the potential to influence their evolution and speciation. Therefore, it turns out that the study of mitochondria is an exciting field that finds many applications, including pest control, and it can shed light on the molecular mechanism of several diseases, improving successful diagnosis and therapeutics. Finally, mito-nuclear co-adaptation, paternal leakage, and kin selection are some mechanisms that can mitigate the impact of the Mother’s Curse.

## 1. Introduction

The symbiotic events that led to the eukaryote and mitochondrial union are considered major innovations for the history of life on Earth and enhanced the eukaryotic evolution as they enabled cells to produce high amounts of energy [1,2]. About two billion years ago, the symbiosis of an alphaproteobacterium, which evolved into the mitochondrion, and a proto-eukaryotic host cell began [3,4]. However, in addition to advances in energy production, this symbiosis also led to the establishment of two genomes within every eukaryotic cell: the nuclear genome and the mitochondrial genome (mtDNA). Following endosymbiosis, a dramatic reduction of the endosymbiont genome occurred because most of the genes were lost or transferred to the nuclear genome [5,6,7]. However, mtDNA managed also to maintain a set of unique genes mainly involved in oxidative phosphorylation (OXPHOS), a process that produces the majority of cellular energy in the form of adenosine triphosphate (ATP) [8]. For this reason, mitochondria are often characterized as the powerhouse of the cell [9]. Oxidative phosphorylation requires the cooperation of five multi-subunit enzyme complexes that are located in the mitochondrial inner membrane. Except for complex II, all of the other complexes are composed of protein subunits encoded by both mitochondrial and nuclear genomes [10]. Thus, a high level of coordination and cooperation is required between nuclear and mitochondrial genes to perform cellular respiration [11]. Taking into account the role of mitochondria in other functions, such as the biosynthetic pathways of amino acids and nucleotides [12], apoptosis [13], cell signaling [14], etc., constant communication between the nucleus and mitochondria is required, and therefore the dysregulation of the mito-nuclear interactions can impose several threats to the cell survival and affect fundamental cellular and molecular processes, that have a negative impact on the fitness of organisms [15,16].

Mitochondria, except for being the energy machines of the cell, also have some intrinsic properties. They are often characterized as semi-autonomous organelles because they can perform their own replication, transcription, and translation [17,18], and mtDNA is present in many copies, ranging from a few hundred to a few thousand for every cell [18,19,20]. Moreover, the mitochondrial genome is a circular, double-stranded DNA molecule of 16.5 kb that has an extreme sequence organization economy [21]. It encodes 11 mRNAs, 22 tRNAs, and 2 rRNAs, but genes have no introns and some of them overlap. Intergenetic sequences are mainly small or lack at all [18,19,21]. However, another interesting characteristic is their high mutation rate, as mitochondria are characterized as being hypermutable, compared to the nuclear genome with which they have to cooperate [20]. This is believed to be due to the fact that mtDNA is continuously replicated, independently of the cell cycle [19,20], and, in contrast to the nuclear genome, although much debate and research are still being conducted, it seems that the mitochondrial DNA recombination can occur, but it is rare [22,23]. According to Muller’s ratchet, asexual populations that are not subject to recombination, accumulate deleterious mutations much faster [24]. Studies suggest that high mutational loads in mtDNA are observed also because of its exposure to reactive oxygen species (ROS), or due to errors occurring during the DNA replication [25]. Finally, it is haploid, and thus, it can be indicated that this, in combination with the small size of mtDNA and its other characteristics, can also contribute to a faster evolution rate. More specifically, in the case of diploidy, the presence of two alleles acts as a protective mechanism. Therefore, when a mutation arises in one allele, the gene’s function is usually not affected as the second allele remains intact and is used as a backup. In contrast, in haploidy, this mutation can be enough to drive evolution and change the gene’s function, favoring adaptation. Experiments in haploid and diploid yeasts also propose that haploidy is associated with a greater susceptibility to single-nucleotide mutations affecting the evolutionary rate [26].

**Figure 1:**
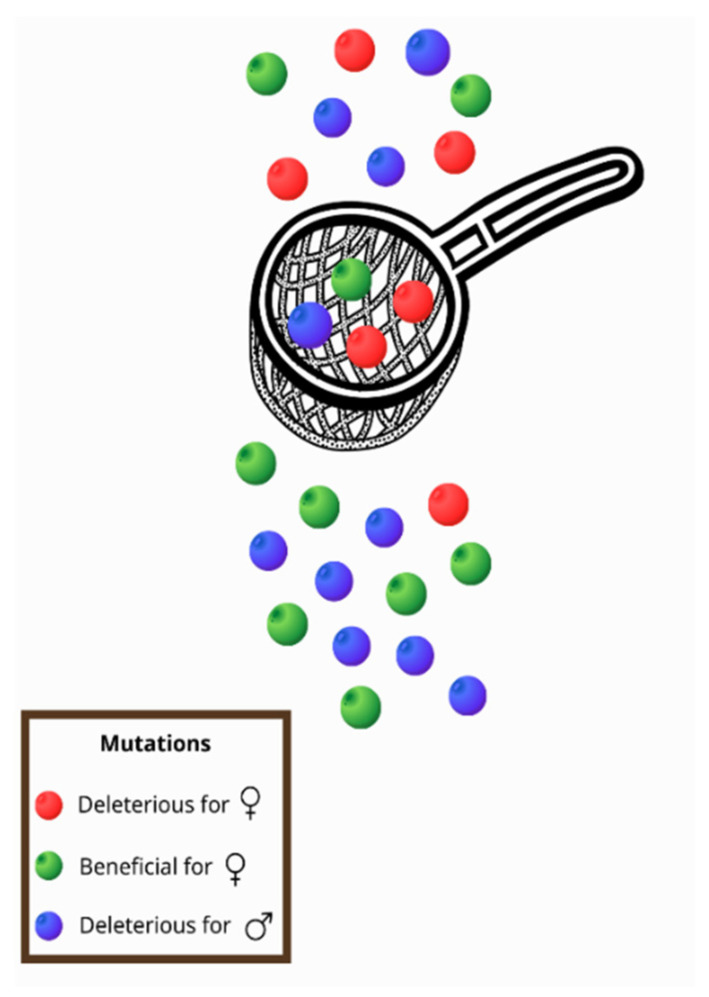
As mitochondria are inherited only by females, a sex-specific selective sieve is created. Thus, mutations that are beneficial for women (green) pass through the sieve, but mutations that reduce female fitness (red) are usually removed. However, in this way, mutations that are deleterious for males (blue) are usually transferred to offspring as they are not subject to selection through females.

All of the intrinsic properties mentioned above and especially the fact that, with a few exceptions, mtDNA is maternally transmitted [22,27], contribute to a phenomenon known as the mother’s curse (MC) or the male mutational load, that can be defined as the accumulation of male-harming mutations in mtDNA [28]. Frank and Hurst (1996) [29] were the first to introduce the idea of the MC in the late 1990s. More specifically, in contrast with the nuclear genome, the mitochondrial genome is inherited from the maternal lineage, and mutations are selected through females [22,27]. Therefore, males can be characterized as an evolutionary dead end for the mitochondrial genome [30]. Mutations that are deleterious for males but neutral, mildly deleterious, or even beneficial for females tend to be maintained in a population, as selection will act on females [31,32,33]. In this way, a sex-specific selective sieve is created (Figure 1) and the deleterious mutations for males accumulate in the mitochondrial genome [28,32,33].

As many years have passed since the first outstanding attempt by N. J. Gemmell et al. (2004) [28] to explore the effect of the MC, today a wealth of data from the existing literature has been accumulated, indicating that the intrinsic characteristics of mitochondria and mtDNA that result in this bizarre phenomenon have the potential to influence our understanding towards different aspects of evolution, population biology, and ecology, as well as biomedical sciences. Therefore, in this review, we aim to shed light on the consequences of the MC phenomenon combined with the requirement for the mito-nuclear cooperation and to provide insight into its implications on a wide range of processes. For this reason, we first summarize the main studies and findings that associate the MC with inherited diseases and examples of reduced fitness in males, including the impact of the curse on male fertility, metabolism, and aging, and also try to explore the association between some of them. In the second part, we investigate its role in ecology and population biology by presenting examples of hybrids’ fate determination through mito-nuclear incompatibilities and the maternal transmission of mtDNA that can even lead to reproductive isolation and speciation, and highlight the important role of the environment in the above processes as the complex genotype-by-environment interactions (G×E) emerge. We also take the opportunity to discuss another more thoughtful side of the phenomenon, as the understanding of all of these mechanisms can be applied to several domains in the future and have implications for medicine and pest control. Finally, in the last part of the article, we consider factors and mechanisms that could mitigate its effect, and as a result, the MC cases can be masked.

## 2. The Bad Luck of Being Male—The MC Is Associated with Diseases That Mainly Affect Male Fitness

Since mitochondria play an important role in many pathways and cellular processes [12,13,14], they are involved in the pathogenesis of many diseases [12,34,35,36], and thus, the accumulation of mutations in mtDNA can have a serious impact on health and fitness, especially for males, due to its maternal inheritance. Therefore, in this section, evidence supporting the association of the MC with diseases is presented, as well as studies that prove its negative impact on male fitness and health status.

### 2.1. Leber’s Hereditary Optic Neuropathy: The First Record of the MC Effect in Humans

For many years, the manifestation of the MC in humans remained only a theoretical possibility, but recently, Milot et al. (2017) [37] provided the first evidence of the phenomenon occurring in humans for a specific mutation associated with a disease known as Leber’s hereditary optic neuropathy (LHON).

By combining the genetic evidence, historical research, and genealogy data, researchers identified a mutation in mtDNA, T14484C, that escaped the natural selection for about three centuries [37]. This mutation is mapped in a gene that encodes subunits of complex I of the mitochondrial respiratory chain and results in the change of a conserved amino acid [38]. Many years earlier, scientists observed that this mutation was the most common in French-Canadian families with LHON, a maternally inherited disease associated with defects in the optic nerve that finally, leads to severe visual impairment [39]. Leber was also the first to report that LHON primarily affects young adult men [40], and nowadays, it is widely accepted that LHON affects males with a higher frequency than females, due to differences in its penetration [41].

This specific mutation causing LHON (T14484C), appears to have been transferred to Quebec by one of les filles du roy (The King’s Daughters), female immigrants who were sent to the colonies between 1663 and 1673 by King Louis XIV [37,42]. The young woman-carrier of the mutation married and gave birth to 10 children, of whom six were women [37]. Milot et al. (2017) [37] assumed that the MC acted on this case and led to the establishment of this mutation, as the data analysis revealed that male carriers of the mutation had a low level of fitness, compared to the females and male non-carriers. In contrast, the female carriers of the mutation had a higher level of fitness, even in comparison with non-carriers. As a result, despite the negative impact on males, for whom the mutation led to a reduced fitness, the frequency of T14484C increased and finally stabilized in the population, due to selection through females. More specifically, this mutation is found in mtDNA and as this is maternally transmitted, the advantage of this mutation for female carriers led to the positive selection and was not removed by natural selection even though it was associated with a lower level of fitness in males. More specifically, this difference in fitness was surprisingly associated with infant mortality, even though LHON usually emerges many decades later in life [37]. In contrast, female carriers had a higher level of fitness, in comparison with female non-carriers, leading to a stabilization of the mutation in the whole population, as they gave birth to more offspring.

It should be noted that in this study, fitness was measured by the individual’s residual reproductive value (RRV) at birth [43]. RRV is a widely used tool in evolutionary and population genetics studies and in simple words, it includes both the current and the expected reproductive output of an individual in the future [44]. In contrast with other measures, such as raw fertility, this methodology has several advantages, such as that it takes into account the age of parents at childbirth and the demographic variation in the population. Then, in the study by Milot et al. (2017), fitness was also adjusted for the population’s growth rate, as in previous studies investigating the historical data of human populations [45], in order to obtain reliable and accurate results.

Now, the frequency of the mutation in today’s Quebecois is surprisingly high as it is found in approximately 1 in 2000 individuals [46]. Therefore, this study provides a new understanding of the MC in humans and proves that it can cause diseases that affect mainly males.

### 2.2. Impact of the MC on the Dimorphic Characters—The Examples of Metabolism and Male Infertility

Sexually dimorphic characteristics are considered those that differ between individuals of different sexes belonging to the same species and they are attributed to evolution through natural selection for the reproductive success [47]. According to the definition of the MC, it is indicated that natural selection can be blind to male-affecting mutations, as selection occurs through females. Therefore, it has been proposed that the main targets of the curse are sexual traits because mutations that affect them have an impact on male fitness and reproductive ability, but generally they have no effect on females or are even beneficial [28,30,33]. In the same way, the selective sieve created by the MC is more likely to act on sexually dimorphic characters [33,48], because the traits that are identical between the two sexes usually function in the same way and mutations affecting them are beneficial or harmful for both males and females. In contrast, mutations that affect male-specific characters and male reproduction are usually neutral for females and more exposed to the MC, continuing to pass onto offspring [30].

The metabolism is considered by scientists to be a dimorphic character, due to the differences between the sexes [48,49]. Females and males exert different metabolic demands [50,51] and as mitochondria, are called the powerhouse of the cell. The mutations on mtDNA usually affect the efficiency of the oxidative phosphorylation and have an impact on the metabolism’s efficiency. Regarding the impact of the MC on metabolism, there is some evidence, but it is limited, as most studies do not make comparisons between sexes or study only one sex [52]. Aw et al. (2017) [49] used flies (*Drosophila melanogaster*) of two different lines, with different mtDNA haplotypes, to test how the different haplotypes affected the two sexes, regarding the mitochondrial function and other measured traits (longevity, fertility, etc.). They observed that, generally, males were more affected than females, and males with a specific mitotype also had lower complex I activity and a higher number of mtDNA copies, possibly as a mechanism to ameliorate the effect of the mutations that led to a decrease in the complex I respiration rate. It should also be noted that the two lines were studied on a constant nuclear background. Moreover, Nagarajan-Radha et al. (2020) [52] tested different mtDNA haplotypes in fruit flies and proved that the MC affected the metabolic rate of males, but had no effect on females. In contrast, Novii et al. (2015) [53] conducted a similar study in the past, but with different results. They tested different mtDNA haplotypes in *Drosophila subobscura* to study their effect on the metabolic rate, but they concluded that although a specific sex effect was observed, it could not be clearly stated that it was male-biased. As we discuss later, this could potentially be explained by the mechanisms developed to compensate for the impact of the MC and thus mask it [53].

Another characteristic example of the effect of the MC on traits associated with different energy requirements is the work of Carnegie et al. (2021) [48], who studied the wing size. Carnegie et al. (2021) [48] used a rather large mito-nuclear panel of nine mtDNA haplotypes and nine different nuclear genomes, resulting in 81 genotypes examined, to test the hypothesis of the MC effect on the wing size, a sexually dimorphic character that is also associated with the standard metabolic rate and body mass in *D. melanogaster* [54]. The researchers finally found that different combinations of mito-nuclear genomes affected the trait studied, but more importantly, they observed a male-biased effect, as mtDNA created a greater variance for males. Thus, they concluded that their hypothesis was proven [48].

In the same perspective, females and males exert different metabolic demands [50,51], and this is reflected intensively in their reproductive systems. The male reproductive system exerts many differences from the female reproductive system and it has higher energy requirements than the females. Mammalian spermatozoa contain many mitochondria in a region called the midpiece, and use ATP for movement to traverse the female reproductive tract and reach the ovum; in addition, ATP is essential for processes required for fertilization, such as capacitation, hyperactivation, and the acrosome reaction [55,56,57]. Therefore, the MC can contribute to male infertility [30,58], as the mutations in mtDNA that alter the ATP production efficiency affect a male’s reproductive ability and, at the same time, have a mild impact on a female’s reproductive system that has lower energy requirements. Innocenti et al. (2011) [33] studied some mitochondrial variants in *D. melanogaster* and observed important differences between the two sexes. More specifically, the variants induced changes in the gene expression of males, affecting approximately 10% of all transcripts, and interestingly, nuclear genes were also affected as the two genomes interact to achieve the proper function of organisms. It was also highlighted that most of the transcripts that were affected, they exhibited a male-biased expression, and they were mainly expressed in the male reproductive tissues, such as the testes [33]. Thus, with this mechanism, it is very likely that the accumulation of the mtDNA mutations affects the reproductive fitness of males. There are also several other studies associating the mitochondrial variants with male reproduction. Dowling et al. (2007) [59] reported that mutations in the cytoplasmic genes affected two traits that are important for a male’s reproductive success in the seed beetle (*Callosobruchus maculatus*): the sperm viability, and length. Other examples that provide evidence for the effect of the MC on the male fertilization capacity can be found in studies involving many different animals. A study on the European brown hare (*Lepus europaeus*) [58] observed that the insertion of a remote population with a different mtDNA haplotype in a captive colony led to a decrease in male fertility, whereas in the rooster (*Gallus domesticus*), researchers found that sperm mobility was affected negatively by the mitochondrial function and a specific mitochondrial variation [60]. In *Drosophila*, some studies also associate the mitochondrial haplotypes with male infertility [61]. As Patel et al. (2016) [62] proved, an mtDNA hypomorph of cytochrome c oxidase subunit II decreases male fertility, but at the same time, it does not affect females. Regarding humans, several studies also associate the variants in mtDNA with an effect on a male’s reproductive ability, leading mainly to a decrease in sperm motility [63,64,65,66,67,68], as it is the main sperm parameter directly associated with large energy requirements.

At this point, the role of the environment that affects the mito-nuclear interactions [69,70] and thus, the MC effect should also be considered. Montooth et al. (2019) [71] tested some flies’ genotypes using different combinations between mtDNA haplotypes and nuclear genomes. They reported that a specific mito-nuclear incompatible genotype caused male infertility but only when the males developed at high temperatures. More interestingly, the phenotype could be saved by alterations in diet, indicating the important contribution of the environment to the MC manifestation. M. F. Camus et al. (2020) [70] also suggested that there is a link between the mtDNA variation, male feeding behavior, and reproductive ability, while Wolff et al. (2016) [72] reported that the environment and specifically the thermal gradient, can play a role in male fertility in fruit flies. Therefore, further research and experiments are strongly recommended to understand the complex interactions between the genotype and environment.

Finally, it should be noted that there may be a particular exception regarding the reproductive investment in some marine species, as there are female fish that produce several thousand or even millions of eggs, indicating an equal or even greater energy investment for reproduction with males. An extreme example is that of a large dolphinfish (*Coryphaena hippurus*) that is estimated to produce approximately 100 million eggs per year [73], but in general, the ovaries are considered very active organs in fish and consume large amounts of energy for the egg production [74]. In these cases, it is possible that the MC does not lead to such a great impact as the mutations that affect the OXPHOS efficiency, and the energy production affects equally females and males, and potentially even greater for females, though it is difficult to estimate with a high accuracy the energy investment for reproduction in both sexes [75]. Therefore, this can potentially prevent the accumulation of such mutations leading to male infertility. However, the number of studies on male infertility in fish species, especially regarding mtDNA mutations, in contrast with studies on mammals, as referred to above, is very limited or even scarce at all, in order to draw reliable conclusions. In conclusion, more research is needed in this particular field to assess the impact of the MC on male infertility for fish species.

### 2.3. MC and the Aging of Males

Aging is a complex process that is associated with some important hallmarks, including the genomic instability, mitochondrial dysfunction, telomere shortening, epigenetic alterations, etc. [76,77]. However, aging is also a process that exerts large differences between the two sexes, since females tend to live longer than males, although they are considered to have a poorer health associated with a greater comorbidity [77,78,79,80]. Taking into account the key role of mitochondria and the mitochondrial genome in aging [81,82,83], as there is an increase in the amount of evidence linking the mtDNA variants with age-related diseases [84], and highlighting the role of mtDNA in the regulation of the lifespan [85,86], several studies indicate that the MC effect can contribute to the longevity gap observed between the sexes.

M. Florencia Camus et al. (2012) [87] screened male and female fruit flies (*D. melanogaster*) with different mitochondrial haplotypes to study the mitochondrial variants and their effect on the longevity traits. They observed that due to the maternal inheritance of mtDNA, numerous mutations in the mitochondrial genome have accumulated, that affect the aging in males but not in females, providing indications for a sex-specific selective sieve. However, the impact of these mutations on the efficiency of the OXPHOS system and in terms of ATP production was not investigated. It is also interesting that Milot et al. (2017) [37], except for proving the MC role in the occurrence of LHON in the Canadian population, among other findings, highlight that the mutation that causes LHON also contributes to a shorter lifespan for men, as it is associated with infant mortality. Wolff and Gemmell (2013) [88], based on previous findings, also indicate that the mtDNA variants and maternal inheritance of mtDNA can lead to the aging asymmetry observed between the two sexes.

Furthermore, following previous studies that show the important role of the environment in the manifestation of male infertility, there is also evidence linking sex-biased aging with environmental factors. Aw et al. (2017) [49] used two *Drosophila* lines with different mitotypes and fed them four diets that differed in the protein to carbohydrate ratios. Then, they tried to explore the mitochondrial genotype-by-diet interactions by measuring four traits, including longevity. They observed an impact of the sex-specific mitotypes, and more specifically, the males with a specific haplotype that ate a specific diet (with higher protein ratios) had a decreased longevity. There are also some other studies indicating that the variation in the mtDNA can have diet-dependent effects on longevity [89,90], but they may not compare the differences between sexes or they can lead to contradicting results, due to the insertion of the new parameters studied. Therefore, no clear conclusions can be drawn about the association between the MC, diet and aging, but all of the above findings highlight the effect of the environment on mito-nuclear interactions and a possible association between the MC and male aging.

Though a comprehensive understanding of the effect of the MC in aging is limited, according to one hypothesis, it can be associated with sexual dimorphism [87] and more specifically, the differences in energy requirements and metabolism between the sexes [30,91]. Males exert a higher basal metabolic rate [30], but since selection occurs through females, the mitochondria with mtDNA mutations have difficulty coping with the higher metabolic demands of males, and therefore are more exposed to dysfunction and damage [30]. Mitochondrial dysfunction is one of the hallmarks associated with aging [76], and thus, can contribute to a different longevity observed between the sexes. However, more evidence is required to support this theory. Furthermore, leaving aside the different metabolic requirements between the two sexes, another link can exist between aging and the MC that goes through metabolism. Studies in many experimental models associate metabolism and energy utilization with the aging process [92,93,94]. More specifically, dietary restriction [95,96], and the availability of specific nutrients, have been correlated with the metabolic alterations and finally an effect on the lifespan [92]. Therefore, as the MC has been associated with an impact on the metabolism, as explained above, maybe this male-biased effect can also negatively affect the longevity of males, due to the metabolism’s contribution, as well as the mitochondrial’s dysfunction to aging.

## 3. MC Can Lead to the Reproductive Isolation between Populations Affecting Speciation Events

The interaction between nuclear and mitochondrial genomes is essential to provide energy in the eukaryotic organisms [97], as discussed previously. However, many times, the metabolic requirements can be affected by exposure to specific environmental conditions, such as thermal and oxygen gradients, favoring adaptive mitochondrial mutations [98,99,100]. The maternal inheritance of mtDNA, though, can have serious implications for populations. More specifically, in this section, it is described how mito-nuclear incompatibility and the MC have been associated with the reduced fitness of intraspecific hybrids in the laboratory and nature, and how they can even lead to speciation affecting the structure of populations. In addition, evidence is presented regarding the role of the environment in the complex interactions involved in this phenomenon.

### 3.1. MC Impact on the Hybrid Breakdown Observed in the Laboratory

Hybridization is characterized as the inbreeding between genetically divergent populations or species that many times results in different types of failure in reproduction [101,102]. Reduced fertility can be observed in F_1_ hybrids, or reduced viability and fitness are present only in F_2_ or later generation hybrids leading to the hybrid breakdown [102]. Hybrids have been studied for many years and Darwin’s observation on hybrid sterility is remarkable [103,104]. However, Darwin also noticed an asymmetry in this pattern, as he stated that “… hybrids raised from reciprocal crosses... generally differ in sterility in a small, and occasionally in a high degree” [104]. Thus, this phenomenon was named Darwin’s corollary and according to this, the reciprocal crosses affect in different degrees the viability and fertility of the hybrids leading to the asymmetric reproductive isolation [103,105,106]. This asymmetry has been observed in many hybridization experiments in a wide range of organisms, as reported by Turelli and Moyle (2007) [104], as well as Bolnick et al. (2008) [106]. More specifically, they refer to examples in insects [107], fruit flies [108], fishes [106,109], plants, fungi, etc. [106]. A characteristic example is a study by Bolnick and Near (2005) [109] on centrarchid fishes, as they observed a hybrid asymmetry in more than 80% of the species pairs tested. Today, there are even more studies presenting cases of such hybrid incompatibilities in the laboratory [110,111,112,113].

**Figure 2:**
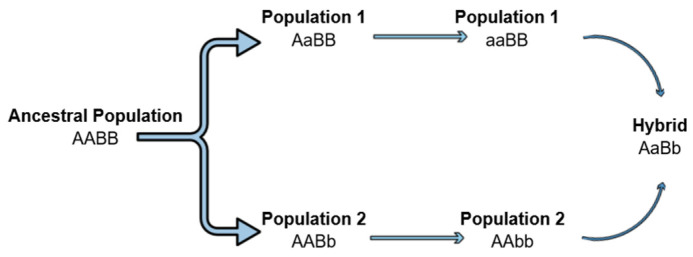
Dobzhansky–Muller incompatibilities can lead to the hybrid breakdown. An ancestral population separated into two lineages. Mutations arise in different loci of the two lineages. Mutations become fixed, and when the two lineages hybridize, a hybrid breakdown is observed as the new alleles can be incompatible.

The hybrid breakdown can be a result of Dobzhansky–Muller incompatibilities (DMIs) as unsuitable epistatic interactions between alleles at different loci occur [106,114,115]. According to this classical model, an ancestral population exists with genotype AABB, but as it splits, different alleles can be fixed into the two isolated populations (AAbb and aaBB). The hybrids produced by inbreeding carry the ancestral alleles (A, B) but also the mutants (a, b). Mutant alleles have evolved in the independent lineages and therefore, it is not clear whether they can cooperate and interact properly, leading to a hybrid breakdown (Figure 2). However, when autosomal loci are involved in DMIs, regardless of the cross direction, all the hybrids will have the same autosomal genotypes (AaBb) and will be affected equally, thus, not explaining the differences in fertility and fitness of the reciprocal crosses hybrids [103,106]. On the contrary, when this asymmetry, observed in the above cases and expressed by Darwin’s corollary is present, it is indicated that the interactive pair contributing to the incompatibilities involves the uniparentally inherited factors, such as mtDNA, chloroplasts, sex chromosomes, etc. [103,105,106].

As explained, the classical DMI model refers to incompatibility between neutral alleles at the two loci described [114]. However, it is indicated that the divergence of populations through DMI sometimes requires selection as a driving force in the presence of the gene flow [116]. In fact, the mito-nuclear DMIs that involve co-adapting loci under a strong selection have been thoroughly studied and their divergent evolutionary trajectories, especially in populations in allopatry, have been implicated in the speciation processes as well [117]. There are several analyses that highlight the role of mito-nuclear DMIs in the organelle dysfunction that leads to the hybrid breakdown and acts as a genetic barrier in the gene flow between the isolated populations [11,118,119]. Following the restriction of the gene flow, each population follows its unique evolutionary path, and the mtDNA functions best in the co-adapted nuclear background, which is optimal for fitness. These interactions have also been studied in laboratory conditions, using xenomitochondrial cell lines, and a common conclusion was the disrupted mitochondrial function of the hybrids that leads to the hybrid breakdown [120,121,122].

Rand et al. (2004) [123] were among the first to turn the focus to mito-nuclear interactions and the intrinsic properties of mtDNA that may play an important role in the above cases. According to their theory, endosymbiont genomes that are uniparentally inherited, such as mtDNA, accumulate mutations at a higher rate, as explained earlier, but these mutations are tolerated because the nuclear genes with which they interact, co-adapt with compensatory mutations to restore the cooperation between the interacting loci (Rand et al., 2004) [123]. As stated in the introduction, a highly coordinated interplay between mtDNA and nuclear DNA is required for the cellular respiration, one fundamental process for cell survival. However, it is hypothesized that in divergent populations, mtDNA is maternally transmitted, while the hybrids will also have the paternal nuclear background that was not co-adapted with the maternal mtDNA, leading to a reduced fitness observed in the reciprocal crosses [123,124]. The first clear confirmation of the above hypothesis came from Ellison and Burton (2008) [124], who observed that the crosses between *Tigriopus californicus* copepods of isolated populations, led to a decrease in the fitness levels of hybrids, due to the incompatibilities between the nuclear and mitochondrial genes. More specifically, hybrids that exerted a low fitness level were then recrossed with maternal and paternal parental lines. Fitness recovery was surprisingly observed only at the backcrossing with the maternal line. Therefore, researchers concluded that since mtDNA is maternally transmitted, this backcross reintroduced the nuclear genome that had adapted with the mtDNA that was transferred to the offspring, leading to fitness restoration [124].

The study by Ellison and Burton (2008) [124] was important because it proved, for the first time, that the maternal transmission of mtDNA and the phenomenon of the MC can affect the fate of hybrids and that the nuclear-cytoplasmic interactions contribute to the hybrid breakdown, laying the foundation for future research. Later studies also attributed the breakdown to particular effects of the mito-nuclear incompatibilities, such as a reduced ATP production [124], a reduced mitochondrial function [125], and elevated oxidative damage [110]. It should also be noted that the MC is associated with several life-history traits, such as longevity and reproduction, as discussed in the previous sections. Therefore, the possibility of such traits’ expressions being involved in the hybrid breakdown, cannot be excluded. Burton and Barreto (2012) [126] also highlighted as a potential mechanism, the mito-nuclear interactions required for the mtDNA replication, transcription, and translation, which are essential for other mitochondrial functions, except for the oxidative phosphorylation, and thus, can have a serious impact on the hybrid’s fitness when disrupted.

### 3.2. The Fate of the Hybrids and the MC: Examples from Natural Laboratories

In addition to all of the examples observed above, it is important to expand the investigation on the MC effect on the hybrid breakdown from the laboratory to the field, which means observations from populations found in the wild.

Hybrids are present in nature and hybrid zones are generally characterized as natural laboratories [127]. The term ’hybrid zones’ refers to the geographical territories where closely related organisms meet and are hybridized [127,128]. They are commonly referred to as natural laboratories for evolutionary biology, because they offer the opportunity for researchers to investigate the evolutionary forces and mechanisms that lead to population disparities [128,129]. Moreover, natural hybrid zones provide more information about the MC phenomenon and its contribution to the hybrid breakdown, since they allow scientists to examine hybrids for many generations and over a long time period, whereas laboratory crosses include a small number of recombination generations [130].

The maintenance of the hybrid zones is attributed to two key processes: random dispersal and the selection against hybrids [127]. However, an interesting fact is that many studies indicate such a large intraspecific variation for many organisms, that boundaries and clear distribution patterns are created in the hybrid zones as the intraspecific populations are not geographically mixed, potentially due to hybrid breakdown [127,129]. These intraspecific phylogeographic patterns and hybrid zones have been well characterized for a variety of organisms in Europe. Bilgin (2011) [131] refers to two distinct patterns observed. According to his observations, for the first pattern, the differentiation of populations, leads to two separate groups, one in Anatolia and the other in the Balkans [131]. This pattern has been noticed in many animals, including the European green toad (*Bufo viridis*), the long-fingered bat (*Myotis capaccinnii*), the killifish (*Aphanius fasciatus*), etc. [reviewed in Bilgin (2011) [131]]. The second pattern observed, also involves two differentiated populations, but the first in this case is found in Western Anatolia and the Balkans, while the second is found in Eastern Anatolia. Examples of the species with the second distribution pattern are the ground squirrels (*Spermophilus* spp.), the mountain frog (*Rana macrocnemis*), the crested newt (*Triturus karelinii*), etc. [reviewed in Bilgin (2011) [131]]. Consistent with these findings, Stamatis et al. (2009) [132] studied also the phylogeography of *L. europaeus* and reported the existence of two distinct clades: the European clade, which includes the largest part of Europe (France, Italy, Greece, etc.) and the Anatolian clade, which includes Anatolia, Cyprus, Israel, and the Greek islands near to the Anatolian coast (Turkey). Between them, a hybrid zone extends to Northeast Greece and Bulgaria [132,133]. Similar patterns are observed for the populations of the same species for a wide range of organisms and in many places throughout the world. More examples can be found in the populations of *Eopsaltria australis* in Australia [134], distinct groups of the marine species *Fundulus heteroclitus*, south and north of the Hudson River in the USA [135], as well as a plant species and, more specifically, a hybrid zone of the *Metrosideros polymorpha* tree on Hawaii Island [136].

Nevertheless, the question that arises is what causes the hybrid breakdown in the above cases, leading to these hybrid zones and the clear phylogeographic patterns mentioned earlier. The phenomenon of the MC and the mito-nuclear incompatibilities, as observed in the laboratory experiments, also appear to play a role in these cases. In an attempt to investigate the distribution pattern of *Lepus europaeus* and the peculiar absence of the Anatolian lineage in Europe, conversely, researchers used two different approaches [11,137]. The analysis of the transcriptomics data from animals of both lineages provided the evidence for co-adaptation between the nuclear and mitochondrial genes of the oxidative phosphorylation machinery [11], whereas the sequencing of the mitochondrial genomes performed in a second study revealed the differentiation of the genes encoding for the mitochondrial OXPHOS subunits between the two clades [137]. Therefore, these results indicate that the hybrid breakdown can be attributed to the MC as the maternal transmission of mtDNA disrupts the cooperation between the nuclear and mitochondrial genes required, negatively affecting the reproductive success of hares coming from different clades.

Furthermore, Morales et al. (2015) [138] studied two geographically distinct populations of the eastern yellow robin (*E. australis*), adapted to different environmental conditions and characterized by two parapatric mitochondrial lineages. They found that the highly differentiated regions of their genomes were rich in genes encoding the mitochondrial proteins involved in OXPHOS, thus suggesting that the mito-nuclear incompatibility can result in the population divergence as the gene flow between them is decreased [138]. Baris et al. (2017) [135] also studied two naturally occurring populations of *F. heteroclitus* found on the south and north sides of the Hudson River with two different mitochondrial haplotypes. They finally observed that the hybrids exerted a reduced cardiac OXPHOS metabolism affected by the mito-nuclear interactions [135].

Thus, all of the above findings imply that, just as in the laboratory, and so in nature, the maternal inheritance of mtDNA and the MC phenomenon can pose a threat to the successful mito-nuclear cooperation affecting the hybrids’ fitness levels, as the OXPHOS function is altered and causes the mitochondrial dysfunction.

### 3.3. MC as a Step Leading to Speciation, the Driving Forces behind It and the Role of the Environment

The driving force behind the mtDNA variation that is established in populations and results in the mito-nuclear incompatibility of hybrids, as discussed above, is the adaptation to different environments. It is widely accepted that the environment is an important regulator of the metabolic state of all living organisms [139]. As the core component of the metabolism is OXPHOS, it seems that the mitochondrial genes and variants found in them play a fundamental role in the adaptation to different environments. There are several studies reporting the variation in the mtDNA genotypes between the populations of the same species that are however, adapted to different environments. Dingley et al. (2014) [140] studied two wild isolates of *Caenorhabditis elegans* derived from England and Hawaii. They observed that the nematodes found in the tropical and warm climate of Hawaii, though belonging to the same species, had a non-synonymous mutation in the mtDNA-encoded *COX1* core catalytic subunit of the mitochondrial complex IV. This mutation had a functional impact, as it altered the energy metabolism, indicating an adaptation to the local environment of Hawaii. As expected, the hybrid of these two populations and the carriers of the Hawaiian mtDNA exerted a decreased lifespan, possibly due to the mito-nuclear incompatibility. Many other studies also associate the mtDNA variation with the adaptation to local environments and altered environmental conditions. Hill (2015) [141] supports this hypothesis and provides many examples of this association between the mtDNA variation and local adaptation, including a non-synonymous mutation in the COX3 core subunit of the mtDNA encoded gene (cytochrome c oxidase subunit 3) of the mitochondrial complex IV that enables bar-headed geese (*Anser indicus*) to fly in low-oxygen environments found at extremely high altitudes, such as the Himalayas. Similar findings in humans have also received attention lately. Chen et al. (2020) [142] propose that a mtDNA variation plays a role in the high-altitude adaptation of native populations in China. Scientists had also observed earlier that differences in the mtDNA sequences of Han and Tibetan populations were associated with an adaptation to high altitudes [143]. These are only a few examples, as there is extensive literature on the mtDNA variations and adaptation to specific environments and environmental alterations [99,99,138,144,145,146].

Adaptation to changing environments is also closely associated with the speciation process. The MC, combined with the need for co-evolution of the nuclear and mitochondrial genomes, can provide a new perspective on the population genetics and speciation, as proposed by Giannoulis et al. (2017) [137]. More specifically, the adaptability of mtDNA to changing environments and its high mutational load, as explained earlier, can lead to the establishment of specific mtDNA mutations in a population. The nuclear genome, however, should also co-evolve and co-adapt for the regular function of OXPHOS. Therefore, in hybridization zones, populations with different genetic backgrounds come into contact. When animals of different populations are trying to reproduce, there is the possibility that the mitochondrial genome transmitted from the mother cannot cooperate with the new nuclear genome, as they have not co-adapted, as indicated in all of the above cases observed in nature and in the laboratory. As a result, hybridization fails and the genetic flow between populations corrupts, leading to reproductive isolation. In time, the natural selection on different genomes and the adaptation of populations to different environments can even lead to speciation. According to the principles of allopatric speciation, cladogenesis begins when two populations are separated spatially or temporally, as the gene flow between them stops. In this case, the mitochondrial-nuclear genome incompatibility, due to the maternal transmission of mtDNA, acts as a barrier that prevents the gene flow, isolating the two populations and acting as a first step leading to speciation. In the absence of the gene flow, populations of the same species are expected to evolve independently. Each population accumulates its own set of adaptations and mutations as the divergence of the mito-nuclear genomes increases. The above hypothesis is further supported by several studies proposing that the mito-nuclear incompatibility plays a role in speciation, observed, among others in lizards [147] and birds [148,149] and has also recently raised the interest of many scientists [117,150].

Though the above observation is extremely important for understanding the processes of adaptation and speciation, it should be noted that future research is required to focus on specific genes that lead to the mito-nuclear incompatibility, hybrid breakdown, and reproductive isolation of populations, as the evidence is limited. In addition, though the above examples lead to the suggestion that the mito-nuclear incompatibilities are associated with speciation, more definitive evidence is required to ensure that no other mechanisms are involved in this process.

It should also be noted, however, that though the role of the environment and adaptation to different environments is very important and it can lead to the mito-nuclear incompatibilities in hybrids, it cannot be considered the only force promoting this incompatibility. More specifically, there is the possibility that the two hybridizing species have acquired mutations during their evolution, independently of the environmental conditions in which they have evolved, that can also cause the OXPHOS dysfunction, possibly due to preventing the proper assembly of the different subunits of the OXPHOS protein complexes and finally leading to the reduction of hybrids’ fitness levels.

## 4. Future Perspectives and Applications

The MC can also be viewed from another perspective, as it has several important implications for future applications and research. These applications hold promise to address global needs and challenges, such as pest management. Furthermore, the use of evolutionary approaches to study genetic diseases could revolutionize this field and improve our understanding of many of them. However, ethical and other considerations arose, based on the prevention of mitochondrial diseases, with the development and application of new techniques in medicine, such as the three-parent technique. Finally, an exception to the rule of the maternal inheritance of mtDNA and the challenge of the mito-nuclear coevolution in bivalves are presented, as this is an interesting field for future investigation regarding the impact of the MC.

### 4.1. MC Application for Pest Management

The MC can have a serious positive impact on human health and agriculture, through its application and use for pest management. The term ‘pest’ refers to organisms that threaten animal and human health, as they transmit various diseases and can also harm crops, livestock, or other important human resources, leading to serious losses to the agricultural sector and the global economy [151,152]. However, despite their negative impact, their control is rather difficult and remains a challenge even today. More specifically, traditional approaches include the use of herbicides, pesticides, etc., but these methods are not cost-effective and, among other disadvantages, they lead to the environmental contamination and the development of resistant insect populations [152,153]. Lastly, pest population fertility control has been proposed as an alternative approach to the traditional methods used, but even in this case, there are many limitations. Today, the sterile male technique (SMT) is considered the most successful of these, as it is an environmentally friendly and non-harmful approach that involves the release of males that have previously been sterilized, in wild populations. When released, sterile males mate with wild females reducing their reproductive capacity. Therefore, after subsequent releases, it is expected that the target population to be locally suppressed [154,155]. There are notable examples of the successful application of SMT [156,157,158], but its main disadvantage is that it is a time-consuming and expensive process that involves the production and release of a large number of insects, thus precluding its use in less developed countries [154].

However, as an alternative approach, and inspired by a fighting strategy from Greek mythology, a few years before, researchers tried to take advantage of the MC for pest control using Trojan females (TFs). Trojan females can be described as female carriers of mtDNA mutations that are benign or even beneficial for females but simultaneously they are harmful to males by affecting their reproductive ability and even lead to reduced male fertility, as discussed earlier. Due to the decline in male fertility, a decline in the size of the population could also be observed. The method that utilizes these Trojan females was named the (TFT) by Gemmell et al. (2013) [159], who first investigated its use and application. In this preliminary study, mathematical models were used to demonstrate that this technique can be used effectively for pest management, highlighting that an obvious advantage is that it can be adapted to be applied to any pest species, since mitochondria are found in all higher organisms [159]. One of the limitations considered in this first study was the concern about the identification of such mutations that are male-harming, but they don’t affect the females’ fitness levels. However, some years later, Dowling, Tompkins, and Gemmell (2015) [160] described a non-synonymous mutation in the mitochondrial cytochrome b gene of the respiratory complex III in *D. melanogaster* that decreases the male reproductive capacity in diverse nuclear backgrounds. This mutation was observed in a naturally occurring population in Brownsville, TX, USA [161]. Wolff et al. (2017) [162] showed that this mutation and TFT can be used for the suppression of fruit fly populations in the laboratory. They inserted Trojan females carrying this mutation in a population and they observed that its size decreased over time while the mutation remained for at least ten generations, avoiding the need for subsequent releases of Trojan females, as required in previous pest management approaches [162].

Though these results are promising for the application of the MC to pest management, this would be a fruitful area for further work, as several questions remain to be answered. First, more male-harming mtDNA mutations with the potential to be used in TFT should be identified in a wide range of organisms, such as insects, mosquitoes, or even rats [162]. The effect of these mutations on the phenotype and on male infertility, as well as their effectiveness in suppressing natural populations and being tested not only in the laboratory, should also be further investigated. Second, as is going to be further discussed in the next section, a better understanding of the mechanisms leading to nuclear genomic variations compensating for the mtDNA mutations is needed and especially the time required for their development. Finally, some years before, the application of the MC for pest management was more a theoretical possibility, limited to some organisms, because it required certain naturally occurring mutations, but today advances in gene editing technologies, even in the mitochondrial genome [163], could facilitate the application of TFT in many species.

### 4.2. Implications and Applications of the MC in Genetic Diseases and Medicine—Ethical Considerations

The first report of the MC in humans in the case of LHON [37], as explained above, can be considered a breakthrough for the field of genetic diseases, and especially for the role of mitochondria in them. The use of evolutionary approaches can also shed light on several other diseases and provide valuable information about their pathogenesis and diagnosis [164]. Furthermore, there are more and more studies suggesting that the MC and mito-nuclear incompatibility may be involved in the pathogenesis of complex disorders [165,166,167]. ‘Mitochondrial diseases’ is a term used for a group of diverse diseases exhibiting a large variability of symptoms, ranging from mild to severe, or even leading to premature death [164,168]. Mitochondrial diseases are usually attributed to the maternal inheritance of mtDNA, the polyplasmy of mtDNA (its existence in many copies in every cell), and the mitotic segregation [167]. However, the study of several other diseases, except for mitochondrial, through the evolutionary lens can also provide evidence for the involvement of mitochondria, improving our knowledge and the chances of a successful diagnosis and therapeutics. A characteristic example is bipolar disorder [165]. For many years, scientists have supported that a mitochondrial dysfunction is one of the main causes of bipolar disorder [169,170]. Among others, the disruption of intracellular calcium signaling, oxidative stress, and redox imbalance have been suggested to be involved in bipolar disorder and psychological disorders, in general [171,172]. Since then, mounting evidence supported this hypothesis but no specific variants were associated with bipolar disorder, explaining the molecular mechanism behind its pathogenesis [173]. Therefore, it was suggested that the mito-nuclear incompatibility, leading to a mitochondrial dysfunction, can contribute to the presence of bipolar disorder, due to a population admixture [165]. As observed in animal populations, in humans, a genetic incompatibility can arise when nuclear and mitochondrial genomes, originating from the different ancestries, accumulate different mutations and, finally, become the underlying force of some diseases [174,175]. Especially in the case of bipolar disorder, there is some more evidence about the role of mito-nuclear incompatibility and the population admixture, but it is contradicting and not enough to strongly support this hypothesis. More specifically, the risk of mental illnesses, including among them bipolar disorder, is higher among admixed individuals reporting two or more ethnic groups [165]. Population admixture was also associated with a higher risk of mood disorders, according to Asdigian et al. (2018) [176]. The study of the mito-nuclear incompatibility could potentially shed light on several other disorders, including autism [177,178], schizophrenia [179], and depression [180], all of which are associated with a mitochondrial dysfunction.

**Figure 3:**
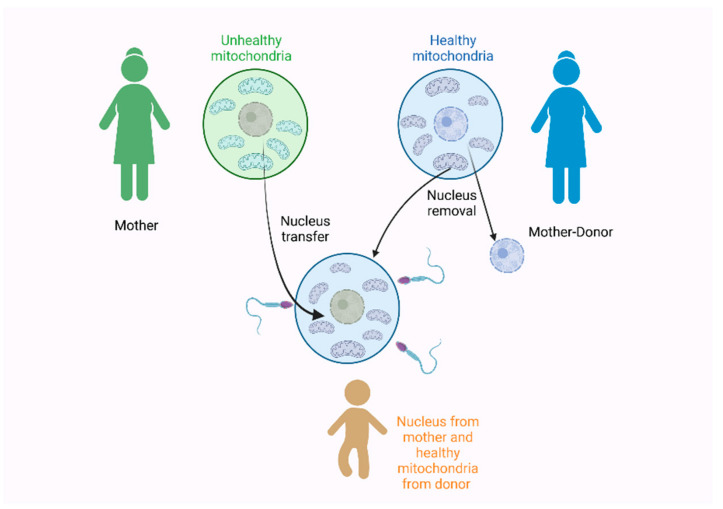
The three-parent technique used in 2016 by Zhang et al. [181] for the prevention of the Leigh syndrome during pregnancy. Figure created with Biorender.com.

All of these findings are also very interesting under the scope of the development of a new technique called the three-parent technique. In 2016, a baby was born by two women and one man to avoid the inheritance of the Leigh syndrome [181]. The Leigh syndrome is an incurable neurodegenerative disorder that affects the mental and motor skills and is associated with a mitochondrial dysfunction. It usually leads to death, due to respiratory failure [182,183]. To prevent the presence of the Leigh syndrome in this pregnancy, the nucleus of the mother’s egg was removed, at first, and transferred to a second egg received from a donor woman. The nucleus was also removed from the donor egg. The final egg contained the nucleus of the mother and the mitochondria of the donor mother was fertilized by the father’s sperm [181], as seen in Figure 3.

The three-parent technique is also considered a mitochondria replacement technique and for many years, it was tested on animals to assess its use for preventing mitochondrial diseases that are inherited, maternally [164]. The greatest scientific concerns were about its safety and efficacy, especially about the possibility of a potential interaction between the nuclear and mitochondrial genomes [164,184]. Furthermore, the risks derived from the micromanipulation of the oocytes and the possibility of a mito-nuclear incompatibility should also be considered [185]. Though the three-parent approach was approved for use in the UK, there is still some conflict as some researchers suggest that long-term consequences should be evaluated and the genetic relatedness between the donor and recipient should also be examined [164]. These concerns are even more important as following the UK example, other countries also considered its approval but without proper legislation [186]. Finally, it should also be noted that, of course, there are ethical issues that also surround these methods described here [185]. More specifically, a serious concern is that the mitochondrial replacement technique can be performed in unfertilized human oocytes but also in fertilized human zygotes, therefore, involving human embryo manipulation [187].

### 4.3. The Mito-Nuclear Coevolution Challenge in Bivalves

A very interesting exception, however, regarding the maternal transmission of mtDNA is observed in bivalves. In bivalves, the general rule of strictly maternal inheritance of mtDNA is violated, since they exhibit a double uniparental inheritance (DUI), where both the parental lineages pass down their mtDNA to their offspring [188,189,190]. These separate molecules, derived from the two parents, often show extreme divergence (e.g., 34% amino acid identity) [191], creating a challenge for the maintenance of mito-nuclear interactions.

However, recent studies have supported the mito-nuclear coevolution in this taxa as well, which was highlighted by a strong correlation in the evolutionary rates among OXPHOS genes encoded by nuclear and mitochondrial genomes [192]. Several authors have proposed different scenarios for the maintenance of both mtDNAs, paternal and maternal, which include, among others, the sex-specific expression of the nuclear-mitochondrial paralogs [193], the conservation of the contact sites between the mitochondrial-nuclear DNA on both lineages [192], and the unequal importance of the paternal and maternal mtDNA for the maintenance of the mito-nuclear interactions, with the maternal ones seeming to be responsible for the interactions, which could explain the increased evolution rates of the paternally inherited mtDNAs [188,194,195,196]. Furthermore, in a recent study, Maeda et al. (2021) [197] studied the coevolution of the genes in bivalves, and their major finding was the action of a relaxed selection in the paternal mtDNA, which indicates that its role is possibly secondary to maintaining the mito-nuclear interaction. Therefore, in the case of bivalves, the coevolution is possibly restricted to the nuclear and maternal mtDNA, while there is less stringent selection on the paternal mtDNA.

This intrinsic case observed in bivalves, could potentially alter the impact of the MC and the accumulation of deleterious mutation for males, due to the uniparental inheritance and the presence of the paternal mtDNA in offspring, but further research is required for the investigation of the MC and its effect in bivalves. This could be a particularly interesting field for future research, as there are still several unanswered questions about the challenge of the mito-nuclear coevolution in bivalves that, combined with the research for the MC, could lead to unexpected and interesting results.

## 5. Why Is the MC Under-Recorded? The Reverse of a Curse

The phenomenon of the MC and the accumulation of deleterious mutations in males can have serious consequences both for the fitness levels of males and for the structure of the populations, as described above. However, it does not appear to be such a widespread phenomenon, as the fixation of male-deleterious mutations could even lead to a decrease in a population’s reproductive ability, endangering its viability [28]. As this rarely occurs in nature, it is suggested that some mechanisms have been developed to alleviate its impact on males and reverse the MC [28,198,199].

At first, the constant interaction between the nuclear and mitochondrial genomes and their co-evolution and co-adaptation is the most studied mechanism that can decrease the impact of the deleterious mutations accumulated, due to the MC on males [200,201]. As explained earlier, the higher mutation rate of mtDNA requires the co-adaptation and co-evolution of the nuclear genome to continue these genomes to cooperate, sustain the mitochondrial activities, and perform fundamental cellular processes, such as oxidative phosphorylation [137,202]. Therefore, when mtDNA mutations arise, the nuclear genome co-adapts to compensate for these mutations, masking the effects of the MC. The most characteristic example in this case is the cytoplasmic male sterility observed in plants. Cytoplasmic male sterility is a phenomenon usually observed in higher plants and usually, the plant does not produce functional pollen [203]. It is a maternally inherited characteristic and is associated with the defects in the mitochondrial genes that lead to a mitochondrial dysfunction. More specifically, the disruption of the mitochondrial membranes, the reduction of the ATP production, and the increase of the reactive oxygen species (ROS), are observed among others [204,205]. Interestingly, however, this situation can be reversed by a nuclear-encoded gene, called the restorer of fertility (*Rf*) [203,206]. Regarding the molecular mechanism involved, according to the studies, the *Rf* gene inhibits the expression of the mitochondrial gene that leads to male sterility, therefore, restoring fertility [206].

At this point, it should be noted that this mechanism does not contrast with the concept described earlier, that natural selection is blind to male-deleterious mutations, as selection occurs through females. It is highlighted that the compensatory mutations arising in the nuclear genome are a result of the constant need for cooperation between nuclear and mitochondrial genomes, in order for the cell to perform the critical functions, such as oxidative phosphorylation [202,207]. Therefore, the decline of male fitness levels could be attributed, in some cases, to such mutations disrupting the interaction required between the nuclear and mitochondrial genomes, but this is a mechanism evolved at first to rescue the interplay between these two genomes [207] and as a consequence, it can also reverse the MC impact and fix the problems created by male-biased mutations. In conclusion, it is not the decrease in male fitness levels that triggers the nuclear compensatory mutations, but the need for cooperation between the two genomes.

Furthermore, paternal leakage is another potential mechanism that can mitigate the effect of the MC. Paternal leakage involves the transmission of the father’s mitochondria to the offspring, leading to heteroplasmy, as both paternal and maternal mitochondria are found in an individual [22]. Heteroplasmy and the indications of paternal leakage, have been observed in several species [22,208,209,210,211], but in general, it is expected to occur rarely in nature, as several mechanisms prevent the transmission of paternal mtDNA (reviewed in [27]). Although there is not enough evidence to support this hypothesis, it is suggested that the paternal leakage is a mechanism developed to reduce the impact of deleterious mutations, due to Muller’s ratchet and the MC [24,208]. However, it should be noted that although some heteroplasmy results have been reported in humans [212,213], the paternal co-inheritance of mtDNA in humans cannot be confirmed [214].

Finally, kin selection can also have a positive impact, reducing the cost of maternal inheritance of mtDNA for males [32,198,215]. Kin selection is characterized as an evolutionary strategy that occurs when an animal self-sacrifices for the benefit of its relatives [216], thus, it can be a mechanism, enabling the adaptation of mtDNA in males [198]. In the case of the MC, male-harming mtDNA mutations would be negatively selected as they harm the fitness of female relatives after their mating [32,199]. Experiments conducted in *D. melanogaster* confirm also that such a mechanism could allow the mitochondrial genome to evolve, reversing the impact of the MC in males [32].

Therefore, all of the mechanisms described above, have the potential to decrease the frequency of male-harming mutations in a population and mask the effect of the MC, but they remain to be fully tested and assessed for their real impact on the accumulation of male-deleterious mutations.

In conclusion, all of these mechanisms described above can explain why, although mtDNA accumulates deleterious mutations at a high rate, it is not widespread in nature, to observe such detrimental male-biased consequences or a decline in populations’ reproduction ability. However, more research is required in this field as it is not very clear what triggers these mechanisms in every case and why some mechanisms are preferred over others. Finally, the real impact of these mechanisms on reversing male-harming mutations and on male fitness should also be explored.

## 6. Conclusions

Mitochondria are important organoids that exert some intrinsic properties, including among them, the maternal transmission of mtDNA. As a result, males are considered an evolutionary dead end and male-deleterious mutations are accumulated, affecting male fitness and reproduction. The MC phenomenon has raised scientists’ attention lately and it is examined from many different perspectives, though there are still many acknowledged gaps.

In this review, we summarize the main findings, regarding the MC in a wide range of fields, including population and evolutionary biology, as well as genetic diseases. Future research will strengthen our knowledge and provide us with valuable information about the potential applications in the important domains, such as pest management or even personalized medicine.

## Data Availability

Not applicable.
